# Findings from computed tomography examinations of Viking age skulls

**DOI:** 10.1038/s41405-025-00309-9

**Published:** 2025-02-18

**Authors:** Carolina Bertilsson, Eva Borg, Maria Vretemark, Henrik Lund

**Affiliations:** 1 Department of Cariology, Institute of Odontology, Sahlgrenska Academy, University of Gothenburg, Gothenburg, Sweden; 2 Department of Oral & Maxillofacial Radiology, Institute of Odontology, Sahlgrenska Academy, University of Gothenburg, Gothenburg, Sweden; 3 Västergötlands Museum, Skara, Sweden

**Keywords:** Oral diseases, Oral anatomy

## Abstract

**Introduction:**

Computed tomography (CT) images can provide information about anatomical structures and pathological processes in ancient skulls. A previous study on the teeth and jaws of 171 individuals in a late Swedish Viking age population, dating around the 10^th^–12^th^ century made clinical examinations that included intraoral radiographs. Current explorative study examined a subset of this population using CT with the aim to investigate if this method could provide additional information about the studied subjects.

**Materials and method:**

The skulls of 15 Viking-era individuals were examined with CT. Two specialists in oral and maxillofacial radiology and one general dentist examined the images together, performing the diagnostics and interpretated the results.

**Results:**

Findings included signs of pathological conditions of the teeth; of the alveolar, mandibular, maxillary and auricular bone; and of the paranasal sinuses and temporomandibular joints. These findings indicated the presence of both clinically detectable conditions, such as dental caries, periodontal disease, periapical destructions and remodelling of the caput mandibulae, but also additional findings such as sclerotization of the mastoid process, infection-induced periosteal bone deposition, and signs of sinusitis.

**Conclusion:**

CT investigation of skeletal remains from an early Christian community in the Viking era in Sweden indicated that the population suffered from numerous orofacial pathologies, including dental disease, sinusitis, otitis, and various infections. The current study, using CT as an investigation method of skeletal remains, indicated that this method could identify conditions that might be difficult to find through ocular inspection. Conclusively, CT is suggested to be an important non-invasive method when used in combination with other examination methods, possibly providing additional information about archeological human remains. Further studies on similar samples are suggested to examine this further.

## Introduction

Osteoarchaeological analysis of human remains comprises a clinical examination under a strong light source by an archaeologist with special competence in osteology. Dental examinations with a dental mirror and an explorer could be part of this examination [1]. This examination is sometimes supplemented with various radiographic methods, two- (2D) and three-dimensional (3D), for deeper examination [2, 3]. 3D imaging using computed tomography (CT) has previously proven to be a valuable retrospective diagnostics tool in paleopathology [3]. It is a non-invasive technique for accessing information on anatomical structures located in inaccessible locations [4]. As a complement to ocular inspection and other examination methods, CT technique can provide information about conditions in areas where other examination techniques does not apply, for example the internal parts of intact crania, and inside undamaged bone tissue. It is especially informative regarding conditions that cannot be observed at a macroscopic level. This technique has previously been used mainly in three areas: reconstructional purposes, diagnostics of pathological conditions and indications of life style, and interpretation of postdepositional stress [5]. The technique has primarily been applied in studies of mummified bodies of humans and animals [6–8], but also skeletal remains [5].

CT was introduced in 1971 [9], primarily for use in clinical medical applications, but has since proven useful in paleopathology. CT images are three dimensional, eliminating the problem of superimposed structures which occurs in conventional radiographic imaging. Use of such imagery in diagnostics, however, should be done by personnel with special training and competence so that information is correctly interpreted.

**Fig. 1:**
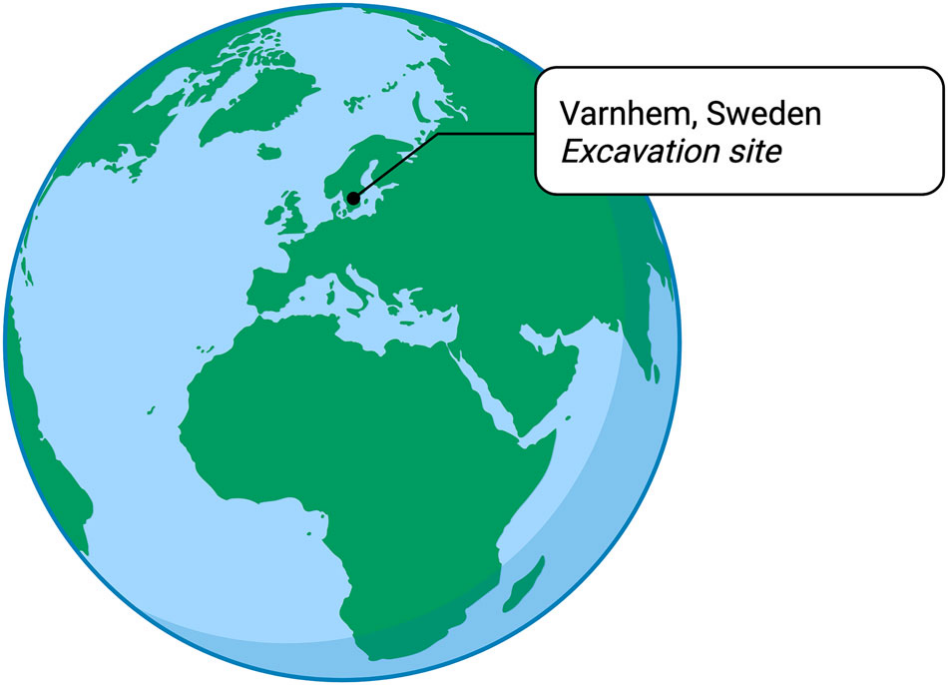
Geographic location of excavation site. Created in BioRender. Lehrkinder, A. (2025) https://BioRender.com/b30x608.

Dating from the 10^th^–12^th^ century AD, and excavated in 2005 on the grounds to the rear of Varnhem Abbey, in Skara municipality, Västergötland County, Sweden, this Viking age population of Varnhem represents one of the earliest Christian settlements in Sweden (Fig. 1) [10, 11] and remains from this burial ground have previously been studied regarding ancestry during the Viking age [12]. The skeletal and dental remains are well-preserved due to the inherent high mineral content of the tissues [1] and the excellent preservational environment of the local burial grounds. Since the remains were generally in good preservation, a lot of information could be obtain from the osteological analysis, performed for around 300 individuals [13]. The jaws and teeth of these individuals were thoroughly examined, giving valuable insights to life during the Viking era [14]. Odontological findings such as dental caries, infections, and other maxillofacial pathologies can provide knowledge about the life of our ancestors [1].

This exploratory study used CT imaging to examine the skulls of 15 Viking-age remains from Varnhem, Sweden with the aim to investigate if this technique could provide additional information about orofacial conditions in the studied individuals.

## Materials and methods

### Study population

Of the 171 partial and complete dentitions that previously had undergone odontological analysis [14], 15 were chosen for a CT examination. The selection was based on convenience sampling, and one author (CB) selected the dentitions based on the following inclusion criteria: (1) cranium with or without mandible and (2) possible to mount in the CT scanner. The exclusion criteria were (1) lack of a cranium with maxillae and (2) not possible to mount in the CT scanner. An osteologist (MV) had already estimated age and sex, for which the method was described in the archaeological report [15]. The study sample comprised 15 individuals, of which nine were male, and six, female. The osteologist determined age at death as ranging from 24 to 60 years.

### Radiographic examination

**Fig. 2:**
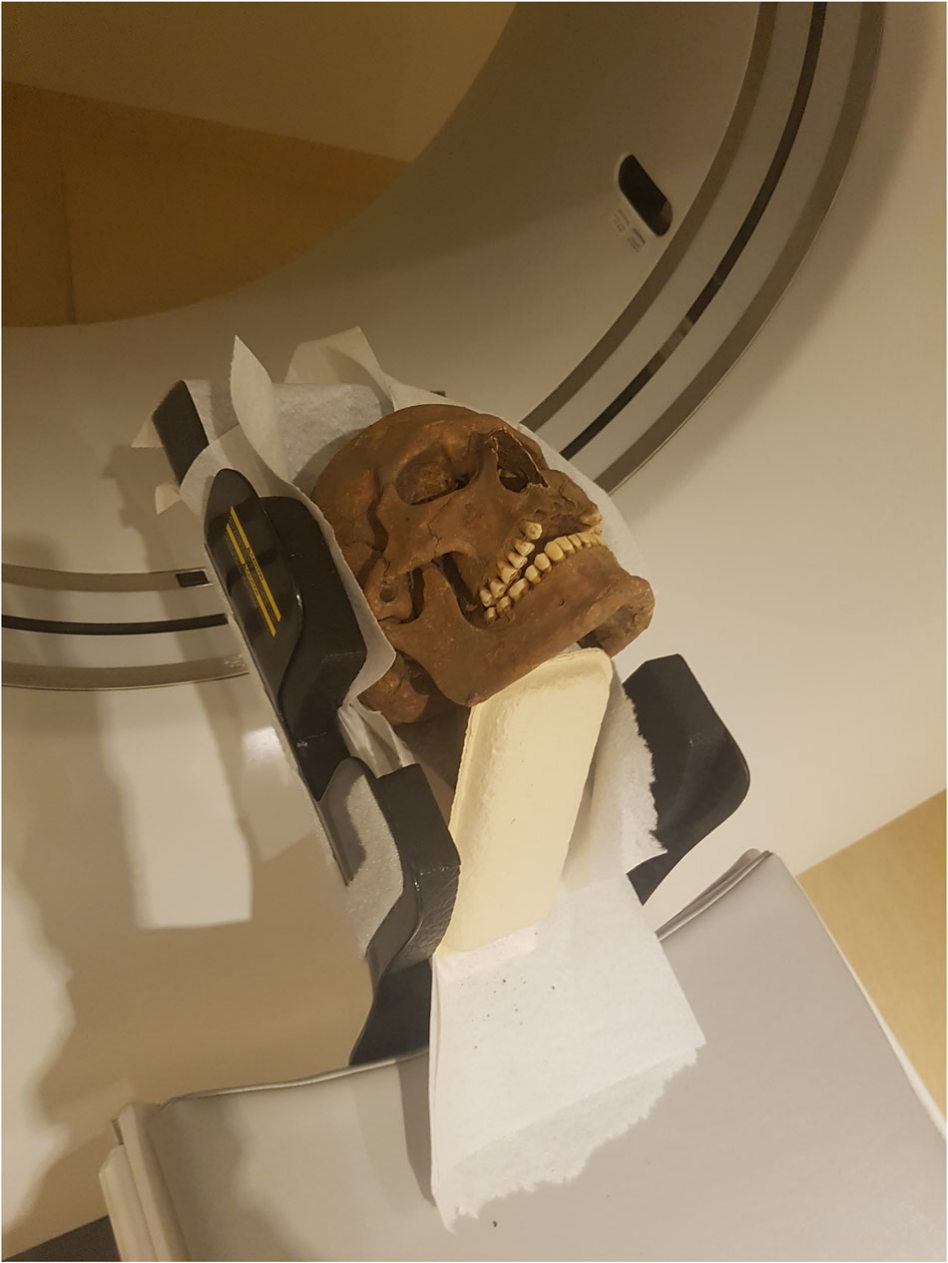
Skull mounted in the CT-scanner.

The CT examination was done using the Optima^TM^ CT660 (GE Healthcare, Sweden AB). A helical scan (0.625 mm slice thickness and interval) of the cranium was made with a field of view (FOV) of 160 mm, and exposure parameters of 80 kV and 50 mA. After examination, an axial stack of images was exported in Digital Imaging and Communications in Medicine (DICOM) format to a medical picture archiving and communication system (PACS; Sectra IDS7; Sectra AB, Linköping, Sweden) for subsequent viewing and interpretation on a high-resolution computer screen. Three dentists, of which two were specialists in oral and maxillofacial radiology with experience in forensic dentistry (EB + HL), and one general dentist with experience in odontological studies of archaeological remains (CB) viewed, recorded and performed the diagnostics together. Figure 2 illustrates the mounting of a skull in the CT-scanner. Viewing and interpretation of the imagery was performed stystematicaly in accordance to the system described by White & Pharaoah [16]. Findings were determined to be of ante mortem (AM) or post mortem (PM) origin, based on the appearance in the radiographic images. Only conditions displaying characteristics of AM processes and conditions were included as AM findings. In case of uncertainty, findings were categorized as PM. Conditions that were a result of taphonomical agents were ruled out as PM defects.

Lost teeth were reported mainly to provide information about the studied material, even though this could easily be studied without CT scanning. All information obtained from the CT scan was reported, including information that could have been obtained by other examination techniques.

### Statistical work

Findings are presented as descriptive statistics.

### Additional information

The current study is a part of an archaeological examination approved by the County Administrative Board in Västra Götaland (Länsstyrelsen Västra Götaland). In compliance with relevant regulations, no additional permits were needed. The remains that were part of this study are located at Västergötlands Museum, Stadsträdgården, Skara, Sweden.

## Results

**Fig. 3:**
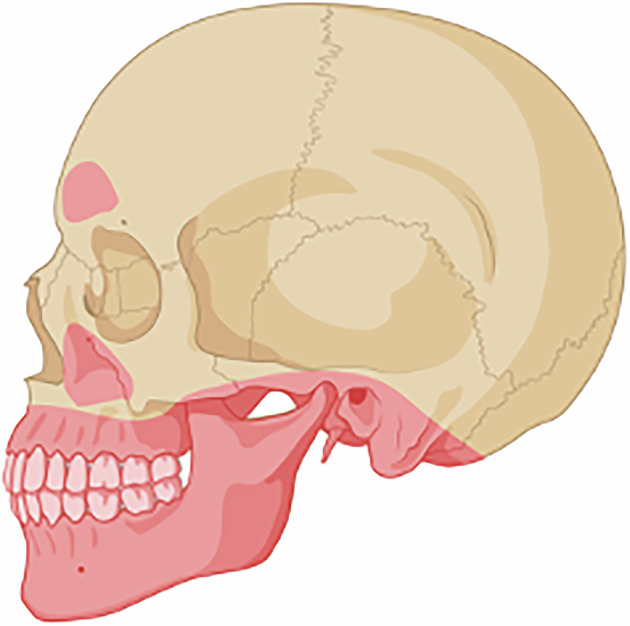
Anatomical sites where pathological conditions occurred. Created in BioRender. Lehrkinder, A. (2025) https://BioRender.com/o64b001.

**Fig. 4:**
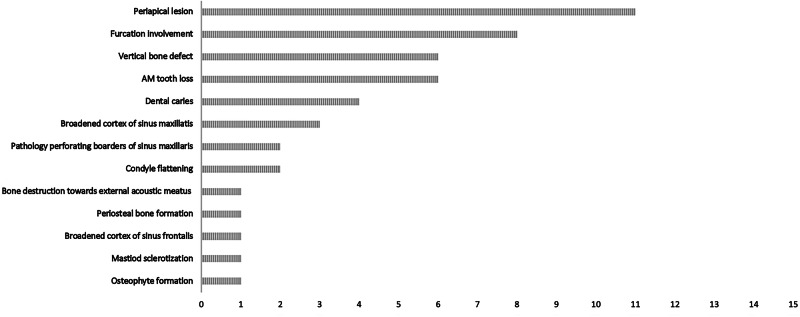
Pathological conditions and numbers of affected individuals.

**Fig. 5:**
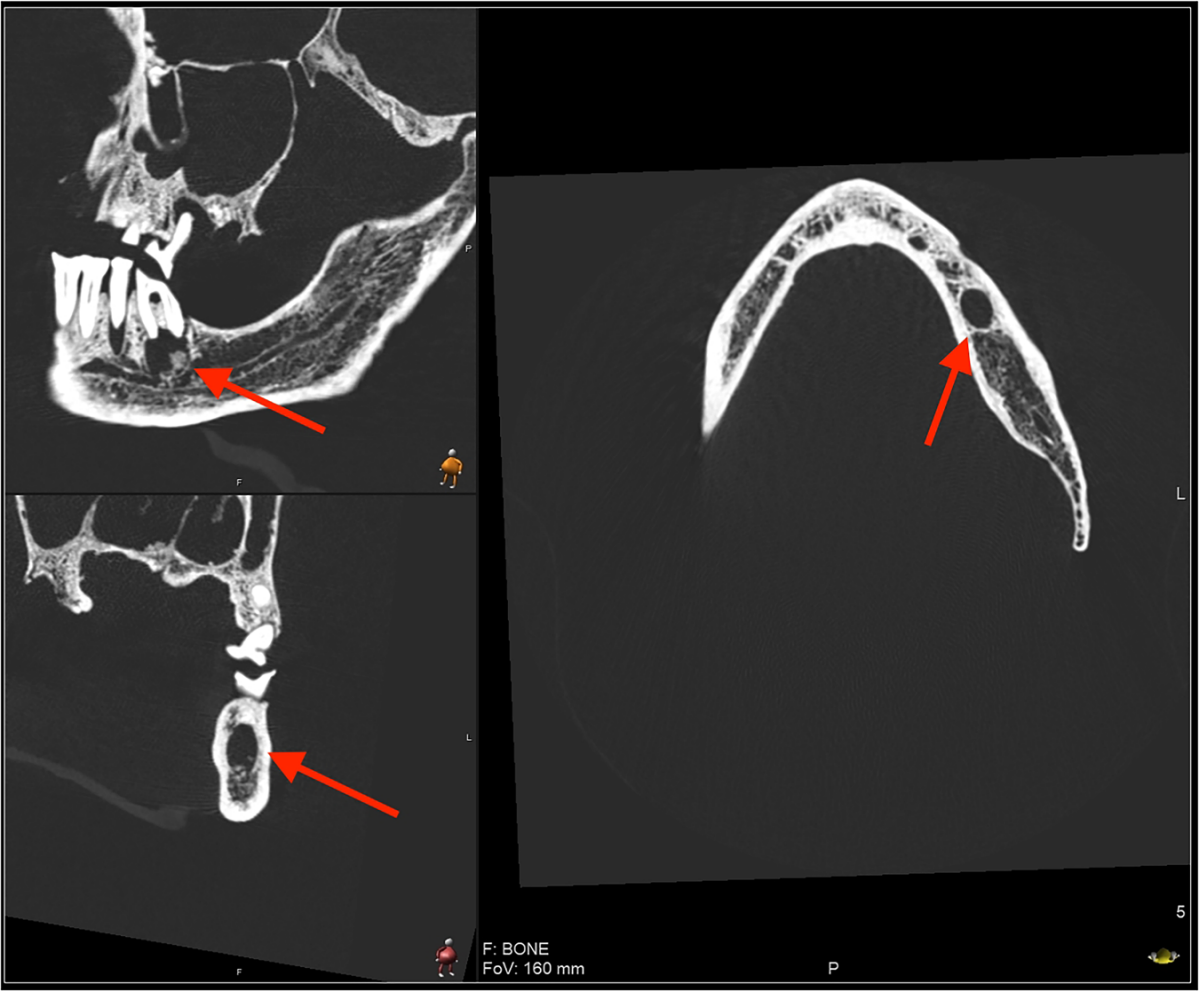
Cyst of tooth 36, individual 12.

**Fig. 6:**
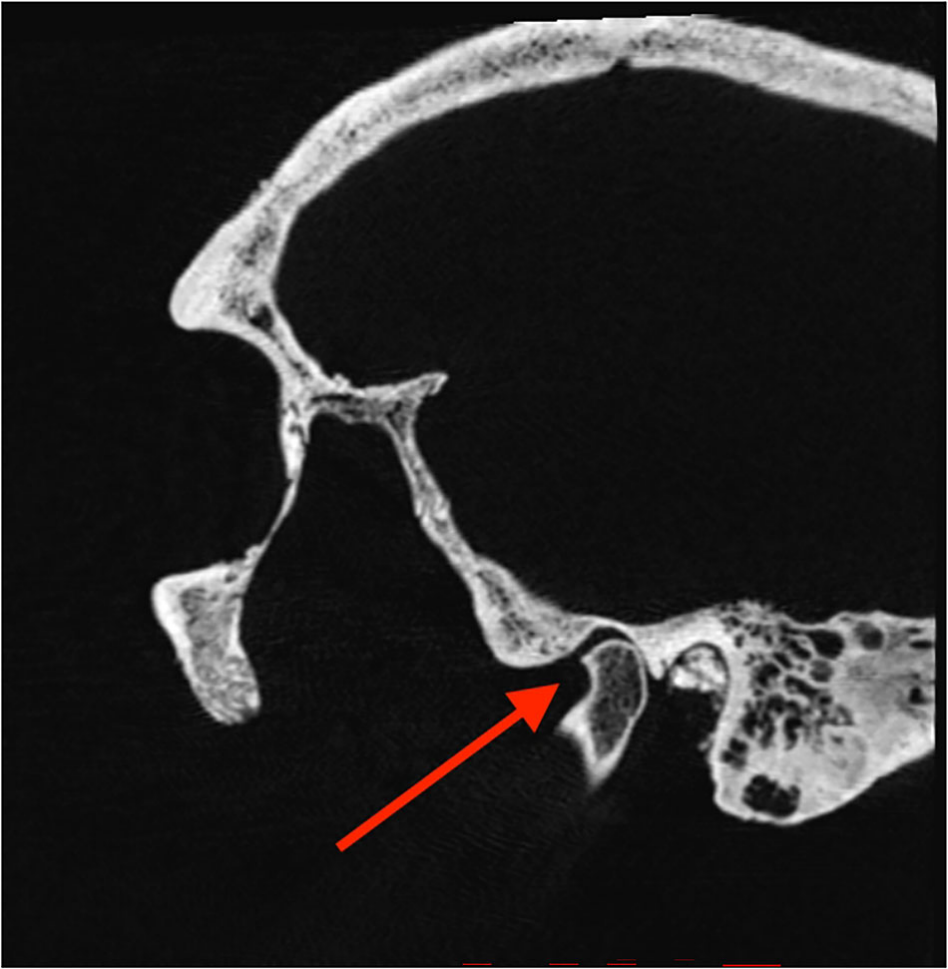
Anterior osteophyte formation of the condyle, individual 12.

**Fig. 7:**
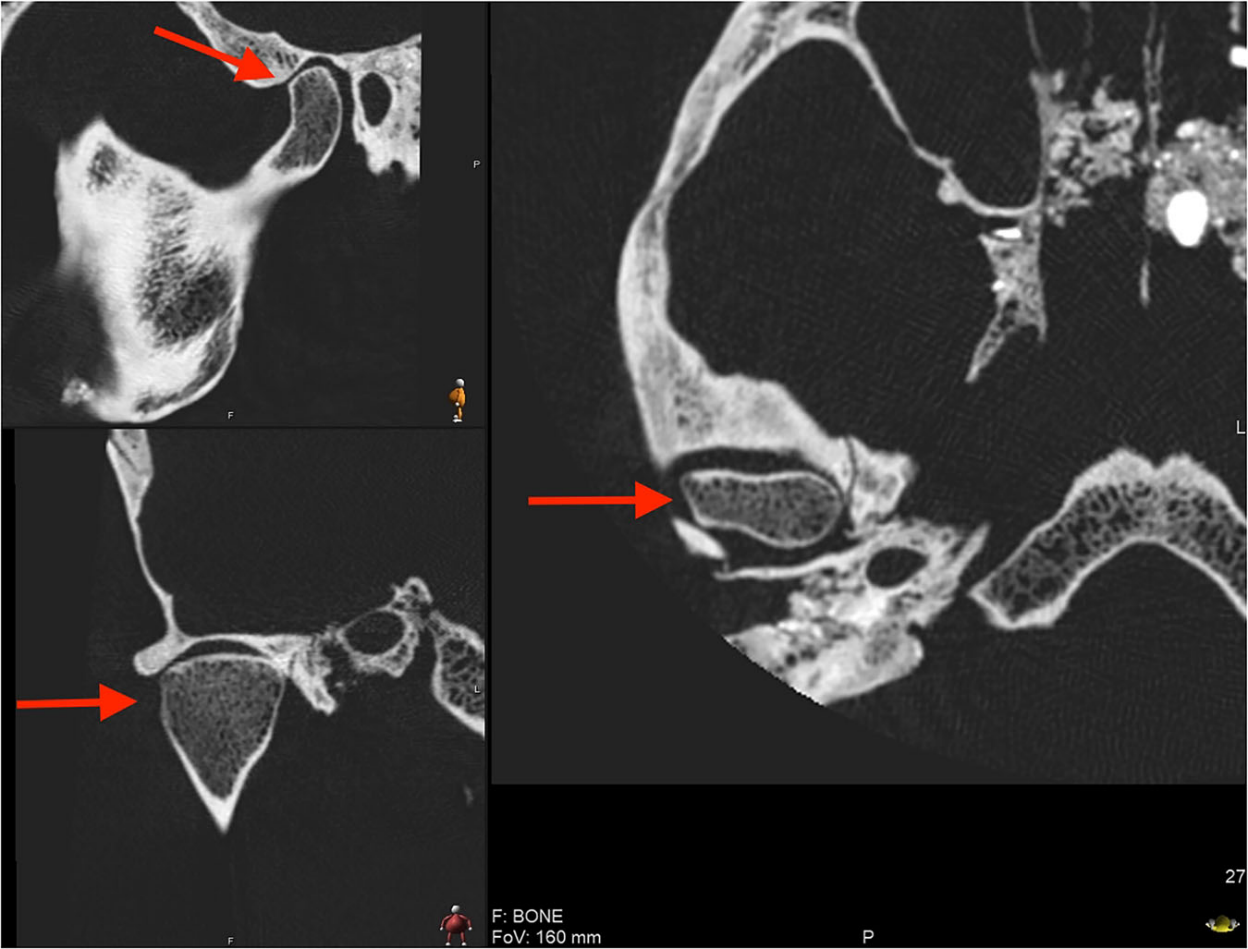
Flattening of condyle, right side, individual 62.

**Fig. 8:**
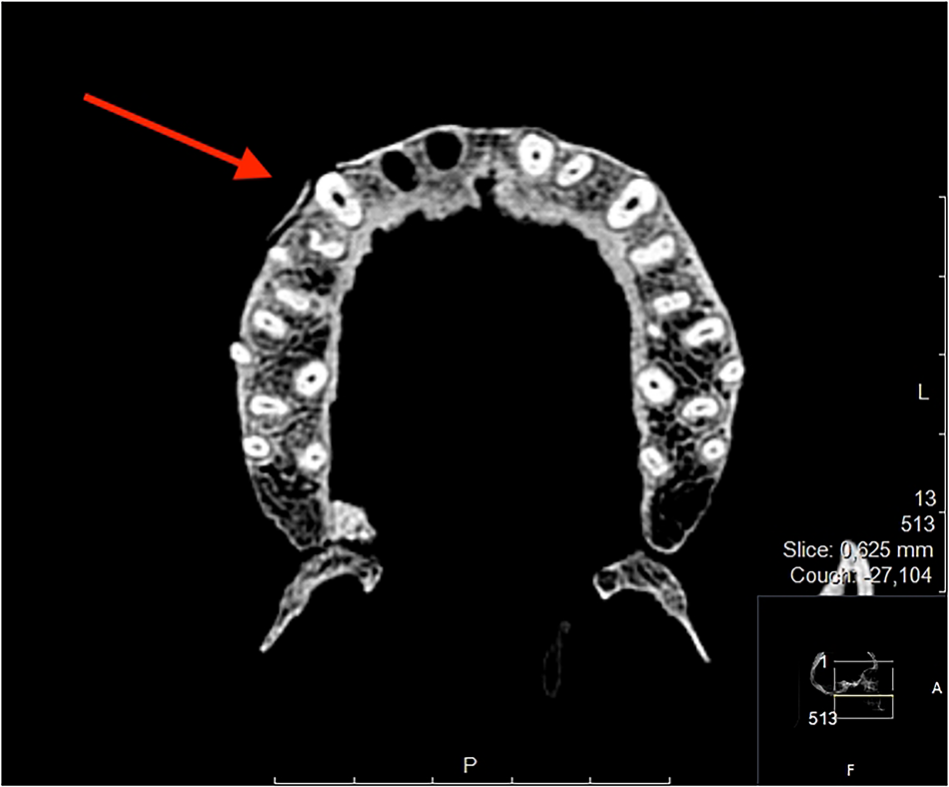
Periosteal bone reaction, individual 98.

**Fig. 9:**
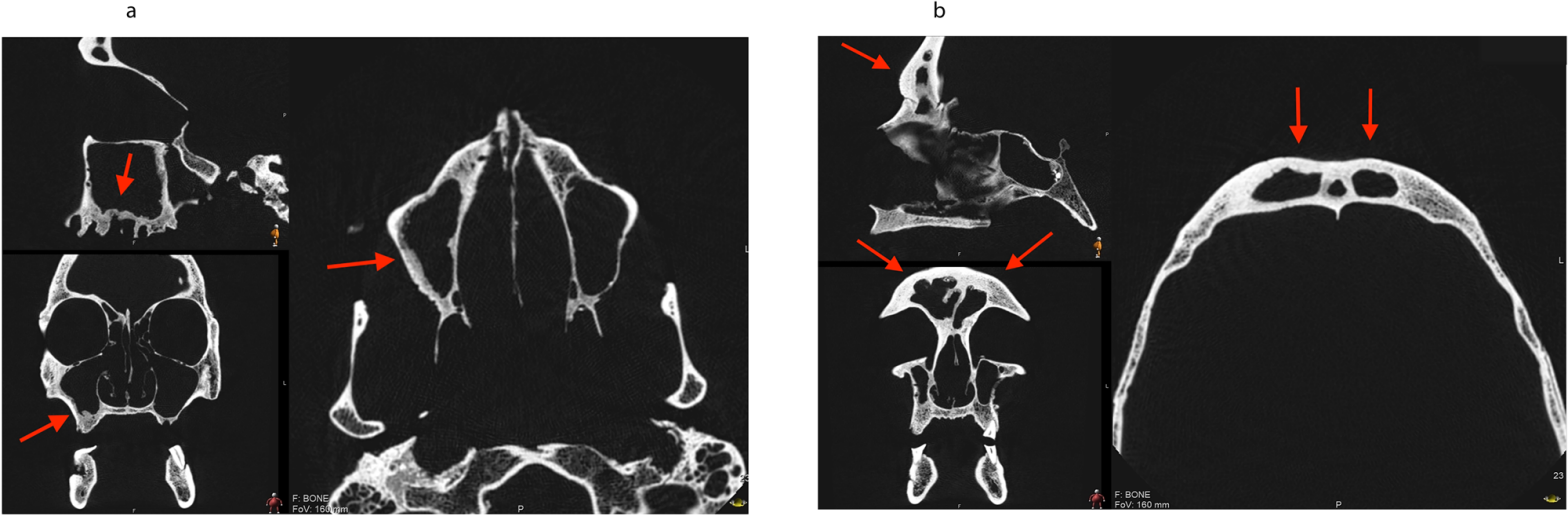
Radiological findings of the sinuses in individual 126. **a** Broadened peripheral cortex of the maxillary right sinus. **b** Broadened peripheral cortex of the frontal sinus.

Indications of several pathologies were found in present study, discussed below. Figures 3 and 4 describe the general results of the CT imaging. Figures 5–9 illustrates some of the findings, such as radiographic signs of pathology of the teeth and alveolar bones, the mandibular, maxillary, and auricular bones, the temporomandibular joints (TMJ), and the paranasal sinuses.

### Lost teeth

With the purpose of describing the study material, number of lost teeth was recorded. All individuals except two (297, 64) had lost teeth post mortem (PM; range 1–22 teeth). During mounting of the skulls in the CT, some teeth were not retained in the alveoli and, thus, not included in imaging (referred to as “lost PM” in this study). Six individuals suffered from ante mortem (AM) tooth loss, including one completely edentulous individual (64), and one individual with an edentulous maxilla (181). The number of teeth lost AM ranged from 1 to 32 per individual.

### Periodontal disease

Radiographic findings of periodontal disease were common in the studied subjects. The findings included horizontal bone loss, vertical bone defects and furcation involvement in two-thirds of the study cohort (10/15 individuals). This was most prevalent in the posterior region, especially in the molars, but the anterior teeth of some individuals had also been affected.

### Periapical inflammatory disease

Signs of periapical inflammatory disease were found in 80% (12/15) of the individuals. The only cohort members without signs of periapical inflammatory disease were individuals 114, 204, and 218. In several cases, the bony borders of the lesions were perforated, creating communication with either the maxillary sinus (individuals 12 and 299), or the oral cavity (individuals 12, 102, 114, 126, 181, 233, and 64). In one case (12) a lesion had diffuse borders. One case of a cyst-like lesion was found (tooth 36, individual 12), illustrated in Fig. 5.

### Temporomandibular joint abnormalities

Findings of abnormalities of the TMJ were common. These findings included osteophyte formation (individual 12, see Fig. 6), flattening of the condyle (62, see Fig. 7, and 144), loss of cortical border (126, 181, and 299), flattening of the articulating eminence (62), destruction zones of varying magnitudes (62, 218, and 299), sclerotization of the bone in the ramus mandibulae (218) and periosteal bone reaction (299). In total, over half of the study cohort (8/15) displayed some variation of TMJ abnormalities.

### Other findings

Other signs of pathological conditions were found, such as carious lesions in 27% of the skulls (individuals 126, 144, 204, 297), retained roots in one individual (233), alveolar bone destruction in 2/15 individuals (181, 218), resorption of the alveolar crest in one individual (126), periosteal bone formation of the alveolar process in one individual (98, see Fig. 8), signs of extensive infection in ramus mandibulae in one individual (218), bone defects in two individuals (62, 233), broadened peripheral cortex of the facial and/or maxillary sinus in concordance with chronic sinusitis in 20% of the individuals (62, 181, 126, see Fig. 9a and b), sclerotization of the mastoid process in one individual (12), and destruction of bone towards the left external acoustic meatus in one individual (299).

Additional findings in this cohort included anatomical variations such as a three-rooted premolar (tooth 24, individual 204), tori palatini (individual 12), and PM conditions such as PM fractures. In several individuals (102, 218, 233), debris trapped in the skull cavities and obscuring the bony structures made interpretation of the images of these segments difficult. Dentine tissue of two molars (teeth 36, 37) from one individual (62) had been sampled PM, thus, causing PM defects.

## Discussion

The usage of radiographic 3D-imaging is important in detecting pathological processes and morphological variations in anatomical locations that are hard to inspect. The technique collects data that provides information on spatial relationships of the examined structures, and enables the interpretator to study the data in any plane, and create 3D reconstructions of both the entire object but also smaller areas of interest [17]. Consequently, it is a useful complement to other examination techniques, such as ocular inspection. Most importantly, radiographic imaging is a non-invasive technique that can provide additional information without affecting the integrity of the studied material. In this study, all findings from the CT scan were presented, however, some of the findings have an increased value since they are not easily studied through other examination methods. These include findings in anatomical areas where access is impossible without destruction of the anatomical tissues, and includes the sinuses, internal auditory organs, the interior part of the crania and skeletal bone tissues, and conditions that does not appear on a macroscopic level.

In this exploratory study, radiographic 3D imaging provided novel information about the health of 15 Viking age individuals from Varnhem. In light of modern medicine and dentistry, this unique information increases our understanding of life in Viking-era Varnhem. The findings indicate that the individuals in this early Christian community may have suffered from numerous orofacial pathologies, including sinusitis, otitis, and infection. These conditions are difficult to detect without the usage of CT technique. Many of these conditions are highly relatable and give a rare insight into the sufferings of these individuals.

Some of the conditions indicated in present study are possible to study without usage of the CT technique, and these include number of lost teeth (AM and PM), periodontal disease, dental caries and some of the TMJ conditions. In some cases, also other pathological conditions related to the teeth and jaws, such as apical periodontitis and cysts, are possible to identify though ocular inspection of the remains. Categorically, this includes conditions that manifest themselves superficially in the bony structures, and in accessible areas easily examined. Even though these conditions could be identified using the CT scan, a radiographic examination is not necessary to determine their presence.

Inarguably, the most important findings in current study are those indicating pathological conditions hardly identifiable using ocular inspection. These findings include signs of infections. In a time when antibiotics, or other modern treatment, were unavailable, these conditions must have been highly challenging. Some infections may have led to death through spreading or sepsis.

Even if the CT technique is highly sophisticated and overcomes some of the limitations of standard radiographs such as super-imposing of structures, there are also limitations to this technique. Since the samples had to be transported to the facility where the CT scanner is located, there is a risk that the skulls were subjected to post-mortem alterations. Also, bone loss and bone defects might occur due to taphonomical agents. The information obtained from CT scanning relies heavily on the interpreter, who must be experienced in using such techniques, understand the technological aspects of the machinery and various modalities, and also be able to differentiate between the radiographic appearance of pathological conditions and taphonomic impact. In current study, this criteria was met since two experienced dentists, specialized in oral and maxillofacial radiology and with a long experience in forensic dentistry, performed the examinations together with a dentist with experience within the field of dental anthropology and oral paleopathology. The low number of included individuals must be considered a limitation in current study, and the findings cannot be applied to the general Viking population. However, the study did provide new insights about the specific studied individuals.

In a majority of the studied remains (12/15 or 80%) the CT images indicated signs of periapical inflammatory disease (apical periodontitis). This condition can sometimes be studied without radiographic examination, but radiographic images usually result in more findings since the process must have penetrated the bony boarders to be detected during ocular inspection. Such pathology could arise from tissue necrosis, a condition caused by inflammatory responses to bacterial invasion of the root canal. Sources of bacterial invasion include dental caries, attrition, and trauma [18]. An earlier study on Viking-age remains from the same population as the sample examined in the present study found high prevalences of dental caries and attrition [14]. Periapical inflammatory disease is not self-healing, and a well-known coeval treatment for these Viking-time individuals was tooth extraction. However, besides extraction, the above study [14] also observed signs of other treatment procedures, such as creating a coronal opening to the pulpal chamber. Untreated lesions might be non-symptomatic, but they can cause pain and intra- or extraoral swelling, and less commonly, infection, airway obstruction, and sepsis, which can be fatal without treatment. The imagery of two of the studied individuals (12, 299) indicated presence of lesions that either had perforated their bony borders and created communication with the maxillary sinus, or displayed a diffuse border towards the same sinus. In another individual (62), the images suggested AM loss of tooth 28 with residual bone destruction and communication with the sinus maxillaris, which could result in sinusitis. The CT also indicated that three individuals (62, 181, 126) in the current study material had a broadened peripheral cortex of the facial and/or maxillary sinus, which indicates chronic sinusitis. Thus, several individuals might have suffered from signs of untreated sinusitis, a condition previously found in other Viking-era individuals [19]. Well-known symptoms of this condition include nasal obstruction, sensation of facial pressure or fullness, nasal discharge and olfactory loss [20], which might have caused difficulties for the affected individuals.

One individual’s radiographic images (12) indicated a sclerotization of the mastoid process, a condition primarily identifyable using radiographic methods, or more invasive approaches. Such findings can be seen in individuals with acute or chronic otitis media [21, 22]. In the acute phase, otitis media may spread from the ear to the mastoid bone [23] causing an infection. Today mastoiditis is treatable, but before the advent of antibiotics, mastoiditis was a cause of death amongst children [23]. The individual displaying this pathology, however, was female and 45–60 years old at the time of death. The CT image of another individual (299), also female and 25–34 years of age at the time of death, indicated a destruction of the temporal bone towards the left external acoustic meatus.

One individual (98) showed signs of a periosteal reaction in the form of bone formation of the alveolar process. This is a radiographic sign of a nonspecific response of the periosteum to irritants or stimuli, such as trauma, arthritis, malignancies, inflammation, and infections, including osteomyelitis [24].

In one individual (12), an osteophyte was located on the anterior surface of the right condyle. Osteophytes are bony outgrowths covered in cartilage, forming at the surface of the articulating bone as a response to arthritic conditions, such as rheumatoid arthritis and osteoarthritis [25]. They are a hallmark radiographic feature of degenerative disease of the TMJ, so it is probable that individual 12 suffered from this condition. In other individuals, other signs of degenerative joint disease were noted including erosion and flattening of the condyle, flattening of the articulating eminence, and destruction zones. These findings would be possible to discover without radiographic imaging, but with the CT scan, the morphology in the interior part of the caput could be studied in detail, providing more information compared to ocular inspection.

Due to the small sample size, the uneven distribution between males (*n* = 9) and females (*n* = 6), and the limited number of remains suitable for this investigation, no conclusions regarding sex differences could be drawn. The entire population, of which the remains in our study are a part, also exhibited a discrepancy in sex ratio. The excavation site mostly comprises the southern grounds of the graveyard, which may explain this. In line with early Christian customs, males were buried south of and females, north of the church [10]. Unfortunately, the fragmented state of many of the remains prohibited the use of radiographic imaging as an addional approach to osteological analysis. Additionally, the extent of PM tooth loss is a source of bias, mostly for determining dental pathologies.

As an investigation method of skeletal remains, this study suggest that computed tomography imagery can indicate pathologies difficult to find through osteological and odontological examination. This method could possibly provide more detailed information about the studied individuals, which is valuable in trying to understand and relate to these Viking-era individuals. This is in parity with the findings of studies on other archaeological remains [26]. Thus, this study suggests the usage of modern methods to be important in the examination of archeaological human remains. However, due to the limited study material, the authors suggest that further studies on similar study materials are essential to provide additional insights regarding the importance of the used technique.

## Conclusion

The findings of the present exploratory study, using CT technique as a compliment to ocular inspection and osteological examination of archeological skeletal remains, although based on a limited number of samples, provided indications of pathological conditions in early Christian individuals in a Swedish Viking era settlement. The findings suggest that CT scanning could be regarded as a valuable non-invasive method, complimenting other examination methods in the study of archaeological remains. Future studies on similar study materials are recommended to provide further insights to the importance of the used technique.

## Supplementary information

Supplementary Table 1

Supplementary Table 1 Legend

## Data Availability

The data that support the findings of this study are available from the corresponding author upon reasonable request.

## References

[1] Hillson S. Recording dental caries in archaeological human remains. Int J Osteoarchaeol. 2001;11:249–89.

[2] Lucas S, Sevin A, Passarius O, Esclassan R, Crubezy E, Grimoud AM. Study of dental caries and periapical lesions in a mediaeval population of the southwest France: differences in visual and radiographic inspections. Homo. 2010;61:359–72. 20813364 10.1016/j.jchb.2010.06.003

[3] Coqueugniot H, Dutailly B, Dutour O. The third dimension in palaeopathology: How can three‐dimensional imaging by computed tomography bring an added value to retrospective diagnosis? Int J osteoarchaeology. 2020;30:538–50.

[4] Uldin T. Virtual anthropology - a brief review of the literature and history of computed tomography. Forensic Sci Res. 2017;2:165–73. 30483637 10.1080/20961790.2017.1369621 PMC6197098

[5] Licata M, Tosi A, Ciliberti R, Badino P, Pinto A. Role of radiology in the assessment of skeletons from archeological sites. Semin Ultrasound CT MR. 2019;40:12–17. 30686362 10.1053/j.sult.2018.10.003

[6] Cramer L, Brix A, Matin E, Ruhli F, Hussein K. Computed tomography-detected paleopathologies in ancient egyptian mummies. Curr Probl Diagn Radio. 2018;47:225–32. 10.1067/j.cpradiol.2017.06.012 28823581

[7] Hoffman H, Torres WE, Ernst RD. Paleoradiology: advanced CT in the evaluation of nine Egyptian mummies. Radiographics. 2002;22:377–85. 11896227 10.1148/radiographics.22.2.g02mr13377

[8] McKnight LM, Atherton-Woolham SD, Adams JE. Imaging of Ancient Egyptian Animal Mummies. RadioGraphics. 2015;35:2108–20. 26562240 10.1148/rg.2015140309

[9] Schulz RA, Stein JA, Pelc NJ. How CT happened: the early development of medical computed tomography. J Med Imaging (Bellingham. 2021;8:052110. 10.1117/1.JMI.8.5.052110 PMC8555965 34729383

[10] Vretemark M, Axelsson T. The Varnhem archaeological research project: a new insight into the Christianization of Västergötland. Viking medieval Scand. 2008;2008:209–19.

[11] Vretemark M. *Christian Vikings in Varnhem*. Skara : Västergötlands museum: Skara], 2018.

[12] Margaryan A, Lawson DJ, Sikora M, Racimo F, Rasmussen S, Moltke I, et al. Population genomics of the Viking world. Nature. 2020;585:390–6. 32939067 10.1038/s41586-020-2688-8

[13] Vretemark M. Aristocratic farms and private churches in the surroundings of Skara in Västergötland – some examples from the 11th to 13th century. *The proceedings of the symposium The Buildings of Medieval Reykholt: the Wider Context, in Reykholt on Iceland, the 18th and 19th of October 2013*; 2017.

[14] Bertilsson C, Vretemark M, Lund H, Lingström P. Caries prevalence and other dental pathological conditions in Vikings from Varnhem, Sweden. PloS one. 2023;18:e0295282. 38091309 10.1371/journal.pone.0295282 PMC10718447

[15] Vretemark M. Osteologisk analys av benmaterial från fornlämning 60, Varnhems sn. Västergöland.: Västergötlands Museum; 2024. Report No.: VGM 2024:60.

[16] Mallya SM, Lam EWN. *White and Pharoah’s oral radiology : principles and interpretation*, 8th edition. edn. St. Louis, M: Elsevier: St. Louis, Missouri, 2019.

[17] Beckett RG. Paleoimaging: a review of applications and challenges. Forensic Sci Med Pat. 2014;10:423–36. 10.1007/s12024-014-9541-z 24682794

[18] Kakehashi S, Stanley HR, Fitzgerald RJ. The effects of surgical exposures of dental pulps in germ-free and conventional laboratory rats. Oral Surg Oral Med Oral Pathol. 1965;20:340–9. 14342926 10.1016/0030-4220(65)90166-0

[19] Sundman EA, Kjellström A. Signs of sinusitis in times of urbanization in Viking Age-Early Medieval Sweden. J Archaeol Sci. 2013;40:4457–65.

[20] Rudmik L, Soler ZM. Medical therapies for adult chronic sinusitis: a systematic review. JAMA. 2015;314:926–39. 26325561 10.1001/jama.2015.7544

[21] Bernatz S, Mahmoudi S, Martin SS, Burck I, Vogl TJ, Ackermann J, et al. Differences in mastoid and middle-ear cavity opacification in CT between intensive care patients and patients with acute mastoiditis requiring surgical treatment. Eur J Radio Open. 2021;8:100365. 10.1016/j.ejro.2021.100365 PMC8227832 34195304

[22] Lee DH, Jun BC, Park JO, Yeo SW. Magnetic resonance imaging of the mastoid cavity and middle ear: prevalence and clinical significance of incidental abnormal findings in a nonotolaryngologic adult and pediatric population. J Otolaryngol. 2006;35:13–18. 16527010 10.2310/7070.2005.4131

[23] Cassano P, Ciprandi G, Passali D. Acute mastoiditis in children. Acta Biomed. 2020;91:54–59. 32073562 10.23750/abm.v91i1-S.9259 PMC7947742

[24] Potter RBBJ. Oral radiology: principles and interpretation by Stuart White and Michael Pharoah. Oral Surg, Oral Med, Oral Pathol, Oral Radiol Endodontology. 2005;99:253–253.

[25] Isberg A. *Temporomandibular joint dysfunction : a practitioner’s guide*. London : Isis Medical Media: London, 2001.

[26] Garvin HM, Stock MK. The utility of advanced imaging in forensic anthropology. Acad Forensic Pathol. 2016;6:499–516. 31239924 10.23907/2016.050 PMC6474549

