# Supplementary material for: Findings from computed tomography examinations of Viking age skulls
Source: BDJ Open. 2025 Feb 18;11:18. doi: 10.1038/s41405-025-00309-9 (PMC11836115; doi:10.1038/s41405-025-00309-9)
Supplement: Supplementary file 1 — Supplementary Table 1 [file 41405_2025_309_MOESM1_ESM.pdf]

Supplementary Table 1 Findings of the computed tomography images: samples, description and raw data. The identities, biological markers, and pathological conditions of the study cohort

Individual number (age in years) gender	Maxilla/ mandibula	Lost teeth (n)	Periodontal disease (tooth number)	Periapical inflammatory disease (tooth number and root affected)	TMJ abnormalities	Other findings
12 (45-60) F	Both	AM: 5<br>PM: 17	FI: 16b	PAL: 16m,p,d , 15 (diffuse border towards s. maxillaris inferior wall), 25 (diffuse lesion), 26mb,dbp.<br>PAL with marginal communication: 26db,p (towards inferior wall of maxillary sinus), 33 (broad destruction zone), 35, 36m,d (cyst-like).<br>Apical sclerosis: 46.	Right: anterior osteophyte formation of the condyle.	Regio 41-31: marginal bone defect.<br>Bilaterally: tori palatini.<br>Right: mastoid process sclerotized.
20 (25-35) F	Both	AM: 0<br>PM: 2	VBD: 28m, b, 37l, 38l<br>FI: 46l	LL: 46d.		
62 (35-50) M	Both	AM: 8<br>PM: 9	FI: 18b, 46b	PAL: 45, 36m,d, 37m,d.	Right: medial flattening of the condyle. Flattening of the articulating eminence.<br>Left: inferior of the condyle joint surface, medially and laterally, small destruction zones.	15 buccal bone destruction, 28 apical bone destruction with communication to the maxillary sinus.<br>48 bone destruction with signs of healing.<br>Maxillary sinus: left, broadened peripheral cortex in concordance with chronic sinusitis.

64 (>50) F	Max	Edentulous maxillae<br>AM: 32<br>PM: 0<br><br>Regions 13 and 23 partially healed alveoli with loss of buccal cortical bone.				Maxillary alveolar crest resorbed.
98 (40-45) M	Both	AM: 0<br><br>PM: 2	FI: 27b	PAL: 13, 23, 46m,d	Right: PM fracture<br><br>Left: PM fracture	Regio 14-12 periosteal bone deposition buccally.
102 (34-45) M	Max	AM: 0<br><br>PM: 2	VBD: 18b, 23m, 24d, 28m<br>FI: 18b, 27 d, b, p	PAL with destruction of buccal cortical bone: 16d,p,m, 26p		
114 (25-30) F	Both	AM: 0<br>PM: 10	VBD: 36m			
126 (45-60) M	Both	AM: 6<br><br>PM: 9	VBD: 47d, 41mdbl, 37d<br><br>FI: 47b, 37b	PAL: 14b, p, 12, 11, 22, 23, 48, 46m,d (with destruction of buccal cortical bone), 41, 36m,d<br><br>Regio 31-33: teeth lost PM with residual PAL.<br><br>Regio 17, 16: teeth lost AM. Residual cyst.	Bilateral: condylar lateral loss of cortical boarder	Caries: 14p(root).<br><br>Regio 27-28 resorbed alveolar crest.<br><br>Maxillary sinus right: broadened peripheral cortex in concordance with chronic sinusitis.<br><br>Frontal sinus: broadened peripheral cortex
144 (24-26) F	Both	AM: 0<br>PM: 4		PAL: 36m,d with marginal communication.	Left: condylar lateral flattening.	Caries: 26o, 36m,o,d,b,l.
181 (45-60) M	Both	Edentulous maxillae<br><br>AM: 30<br>PM: 2		PAL: 32 with buccal perforation of cortical bone.	Left: condylar lateral loss of cortical border.<br><br>Right: condylar lateral and medial loss of cortical border.	Generally resorbed alveolar crest.<br><br>Regions with destruction of the buccal/lingual bone: 21, 23, 47, 45, 43, 33, 37.

						Maxillary sinus bilaterally: broadened peripheral cortex in concordance with chronic sinusitis.
204 (30-50) M	Max	AM: 0<br>PM: 1				24 three roots.<br>Caries: 25d(3).
218 (25-35) M	Both	AM: 0<br>PM: 5	VBD: 17d, 26m, b, 36d		Left: general destruction of condyle.	Regio 24-25 loss of buccal bone cortex.<br>Sclerotization in the buccal region of the ramus mandibulae (right side).
233 (45-60) M	Max	AM: 3<br>PM: 1	FI: 18b, 27b, d, 28b, d	PRL: 16mb,db, 26db.<br>PAL: 16p(with buccal cortex destruction), 21, 22, 23, 24b,p, 26p (with cortex destruction), mb.		Regio 16, 25: retained roots.<br>Regio 14: destruction of buccal cortical bone.
297 (20-25) M	Both	AM: 0<br>PM: 0	VBD: 26m, 27d, 47d<br>FI: 27d	PAL: 26m,b		Caries: 46 occlusal.<br>48 rotated mesially.
299 (25-35) F	Both	AM: 0<br>PM: 22		PAL: 18m, d with destruction of maxillary sinus inferior border.	Right: loss of lateral cortical border of the condyle<br>Left: Lateral bone destruction of the condyle with periosteal bone reaction.	Regio 23-25: PM buccal alveolar bone fracture.<br>AM bone destruction towards the left external acoustic meatus.

Abbreviations

TMJ= Temporomandibular joint

F= Female

M=Male

AM= Ante mortem

PM= Post mortem

FI= Furcation involvement

PAL= Periapical lesion

VBD= Vertical bone defect

LL=Lateral lesion
PRL=Periradicular lesion
RC= Right condyle
LC= Left condyle
m= mesial
d= distal
p= palatal
b= buccal
l=lingual
