# Supplementary material for: Findings from computed tomography examinations of Viking age skulls
Source: BDJ Open. 2025 Feb 18;11:18. doi: 10.1038/s41405-025-00309-9 (PMC11836115; doi:10.1038/s41405-025-00309-9)
Supplement: Supplementary file 2 — Supplementary Table 1 Legend [file 41405_2025_309_MOESM2_ESM.docx]

Supplementary material

**S1 Table: Findings of the computed tomography images.** Samples, description and raw data. S1 presents the identities, biological markers, and pathological conditions of the study cohort.
